# LOW-NICKEL DIET AS A STRATEGY IN THE TREATMENT OF *HELICOBACTER PYLORI* INFECTION

**DOI:** 10.1590/S0004-2803.24612024-108

**Published:** 2025-07-21

**Authors:** Beatriz Soares BRITO, Felipe Ferreira Ribeiro de SOUZA, Daniel Machado BAPTISTA, Rafael Bandeira LAGES, Ricardo Correa BARBUTI, Tomás NAVARRO-RODRIGUEZ

**Affiliations:** 1 Universidade de São Paulo, Departamento de Gastroenterologia e Hepatologia, São Paulo, SP, Brasil.

**Keywords:** Diet therapy, *Helicobacter* infections, Helicobacter pylori, nickel, proton pump inhibitors, Dietoterapia, infecções por *helicobacter*, helicobacter pylori, níquel, inibidores da bomba de prótons

## Abstract

**Context:**

The rising antibiotic resistance in *Helicobacter pylori* (*H. pylori*) infection have made treatment more challenging. A low-nickel diet may improve eradication rates based on the bacteria’s mechanisms.

**Objective:**

This study aimed to evaluate the effect of a low-nickel diet during standard triple therapy on the rate of *H. pylori* eradication.

**Methods:**

This randomized clinical trial included patients with *H. pylori* infection who were classified into the following two groups: low-nickel diet and regular diet. Both groups received the standard triple therapy with amoxicillin, clarithromycin, and a proton pump inhibitor. After at least 8 weeks of treatment, a control test for *H. pylori* was performed.

**Results:**

Per-protocol analysis showed a higher rate of *H. pylori* eradication in the low-nickel diet group than in the regular diet group (91.7% vs 75.8%; *P*=0.026). In addition, obesity was associated with poorer eradication rates (73.2% vs 88.9%, *P*=0.038). Patients on a low-nickel diet were 3.41 times (1.21-11.40) more likely to have *H. pylori* eradication than those on a normal diet.

**Conclusion:**

This study showed that low-nickel diet, which is a low-cost and low-risk intervention, may be an appropriate strategy for increasing *H. pylori* eradication rates.

## HIGHLIGHTS

• Evaluate the effect of low-nickel diet during standard triple therapy on the rate of *Helicobacter pylori* eradication.

• This randomized clinical trial included patients infected with *Helicobacter pylori* in groups with a low-nickel nickel or regular diet, who underwent standard triple therapy and the efficacy of bacterial eradication was compared between both groups.

• Per-protocol analysis showed a higher rate of *Helicobacter pylori* eradication in the low-nickel diet group than in the regular diet group (91.7% vs 75.8%; *P*=0.026).

• The low-nickel diet is an appropriate strategy for increasing *Helicobacter pylori* eradication rates.

## RESUMO

**Contexto:**

O aumento das taxas de resistência aos antibióticos na infecção por *Helicobacter pylori* (*H. pylori*) tem tornado o tratamento desafiador. Uma dieta pobre em níquel pode melhorar as taxas de erradicação com base nos mecanismos da bactéria.

**Objetivo:**

Este estudo teve como objetivo avaliar o efeito de uma dieta pobre em níquel durante a terapia tripla padrão nas taxas de erradicação do *H. pylori*.

**Métodos:**

Este ensaio clínico randomizado incluiu pacientes com infecção por *H. pylori*, classificando-os em dois grupos: dieta pobre em níquel e dieta padrão. Ambos os grupos receberam terapia tripla padrão com amoxicilina, claritromicina e um inibidor da bomba de prótons. Um exame de controle de cura do *H. pylori* foi realizado após pelo menos 8 semanas do tratamento.

**Resultados:**

A análise por protocolo revelou uma taxa de erradicação mais alta no grupo da dieta pobre em níquel em comparação ao grupo da dieta padrão (91,7% vs 75,8%; *P*=0,026). Além disso, a obesidade estava associada a taxas de erradicação mais baixas (73,2% vs 88,9%; *P*=0,038). Pacientes em uma dieta pobre em níquel tinham 3,41 vezes mais chances (1,21-11,40) de erradicação do *H. pylori* em comparação aos que estavam em uma dieta padrão.

**Conclusão:**

Este estudo sugere que uma dieta pobre em níquel, intervenção econômica e de baixo risco, pode aumentar as taxas de erradicação do *H. pylori*.

## INTRODUCTION

*Helicobacter pylori* (*H. pylori*) is responsible for causing one of the most common chronic bacterial infections worldwide. This infection is associated with dyspepsia, gastroduodenal ulcer, chronic active gastritis, gastric cancer, and lymphoma (mucosa-associated lymphoid tissue)^1^. *H. pylori* eradication is directly related to the natural history of these conditions, particularly reducing the incidence and complications of peptic ulcer disease, gastric adenocarcinoma, and lymphoma. However, the increase in antibiotic resistance rates has made eradication more challenging^2^, highlighting the need to develop new strategies to increase the probability of therapeutic success.

According to the Sixth Maastricht Consensus^3^ and the Fourth Brazilian Consensus^1^, therapy with amoxicillin, clarithromycin, and proton pump inhibitors (PPIs) for 14 days is still the first-line option in the absence of assessment of antimicrobial susceptibility and is contraindicated when the primary resistance to clarithromycin is greater than 15-20%. A previous study conducted by our group revealed a resistance rate of approximately 8%^4^. A more recent national multicenter study, which did not include São Paulo, showed a rate of molecular resistance to clarithromycin in 16.9% of the population studied^1^ ^,^ ^5^, raising concerns about the possible inefficiency of the conventional first-line treatment. The use of quadruple therapies appears to be an important option; however, although still effective, they pose a greater risk of adverse effects, especially regarding the intestinal microbiota and the increased resistance of other bacteria^6^. Quadruple regimens that include bismuth have become limited due to the reduced availability of this component in the national territory of Brazil^1^.

The decrease in therapeutic efficacy has been attributed to factors, such as low adherence to treatment, smoking, genetic polymorphism of CYP2C19, obesity, and other comorbidities, in addition to previous use of antimicrobials^7^ ^-^ ^9^. In addition, dietary modifications may be related to variations in *H. pylori* eradication rates, especially regarding the reduction in the intake of nickel-rich foods^10^.

**FIGURE 1.**
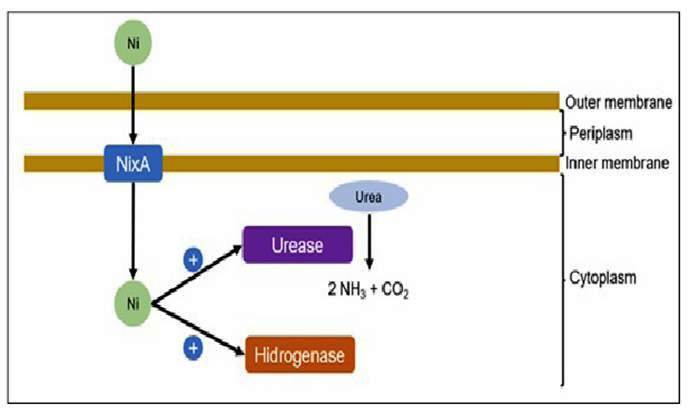
Representative schematic of the relationship between urease and hydrogenase activity and nickel metabolism. Nickel is transported into the *Helicobacter pylori* cytoplasm via the NixA transporter. Once inside the cell, nickel activates both urease and hydrogenase enzymes, leading to the production of nitrogenous compounds and enhancing the bacterium’s resistance to gastric acidity.

Nickel acts as an essential cofactor for some virulence mechanisms present in pathogens, acting as a decisive component for *H. pylori* colonization and chronic infection. Among these mechanisms, the synthesis of Ni-urease and Ni-hydrogenase enzymes stands out. Urease comprises up to 10% of the total *H. pylori* proteome and has a crucial catalytic function through which it converts urea into carbon dioxide and ammonia, which constitute an essential source of nitrogen for microorganisms, in addition to contributing to the neutralization of the cytoplasmic pH of the pathogen (Figure 1). Additionally, urease protects bacteria against oxidative stress. Hydrogenases also have an important catalytic function through which they oxidize hydrogen and generate energy through ATP formation in cells^11^ ^,^ ^12^.

Thus, it is hypothesized that a low-nickel diet may reduce the colonization potential of *H. pylori* and increase its susceptibility to treatment with antimicrobials^10^. 

This study aimed to evaluate the effect of dietary nickel restriction during standard triple therapy on the rate of *H. pylori* eradication.

## METHODS

This randomized clinical trial was conducted between 2017 and 2021 and included 143 patients treated at the Outpatient Clinic of the Stomach Discipline of the Department of Gastroenterology, University of São Paulo School of Medicine, Hospital das Clinicas. The inclusion criterion was the presence of *H. pylori* confirmed using an invasive method (urease or histological test), regardless of the clinical presentation. The exclusion criteria were as follows: age <15 and >90 years; nickel allergy; chronic use of aspirin or nonsteroidal anti-inflammatory drugs; use of antibiotics or chemotherapy within four weeks before the study; important structural alterations, such as pyloric stenosis or complicated gastroduodenal ulcers; and severe comorbidities, such as decompensated hepatic, cardiac, respiratory, or renal failure, in addition to any comorbidity that could contraindicate endoscopic examination under adequate sedation.

After obtaining informed consent, the patients were randomized to either the intervention or control group. For all patients, a seven-day treatment was performed with the conventional triple regimen consisting of amoxicillin 1 g, clarithromycin 500 mg, and PPI (lansoprazole 30 mg, esomeprazole 20 mg, or omeprazole 20 mg) orally and twice a day, in accordance with the recommendations of the main consensuses of the time^13^ ^,^ ^14^. The study began in 2017, when a seven-day regimen was still the standard recommendation. In the intervention group, participants received dietary recommendations and were instructed to avoid foods with high-nickel content throughout the 7-day antibiotic treatment period as shown in Table 1. There was no restriction on the use of nickel-containing utensils during the specified period. In the control group, dietary restrictions related to nickel consumption were not recommended.

**TABLE 1:** Types of food with low and high nickel.

Type of food	Low nickel food	High nickel food
Fruit	Peach, pear, banana, blueberry, strawberry, blackberry Obs: only fresh or cooked	Raspberry, pineapple, fig, date, plum
Vegetables and legumes	Peppers, cucumber, eggplant, cabbage, cauliflower, bok choy Obs: only fresh or cooked	Green and leafy vegetables: spinach, kale, lettuce, beans, soybeans, lentils, chestnuts, walnuts
Dairy	All dairy products: milk, cream cheese, butter and yogurt	Chocolate milk, raspberry yogurt and citrus
Animal protein	Chicken, turkey, beef and eggs	Shrimp, oysters, salmons. Canned meats and fish like tuna.
Grains	Refined wheat, pasta, white rice, corn cereal, common bread	Whole foods: whole wheat, multigrain flour, oats, brown rice, sunflower seeds and sesame
Drinks	Coffee, tea, soft drinks, fruit juices low in nickel	Citrus drinks like citrus and apple juice, chocolate drinks

After at least eight weeks of treatment, *H. pylori* eradication was evaluated using histological methods, labeled urea breath test, or fecal antigen test, according to the availability and clinical indication.

**FIGURE 2.**
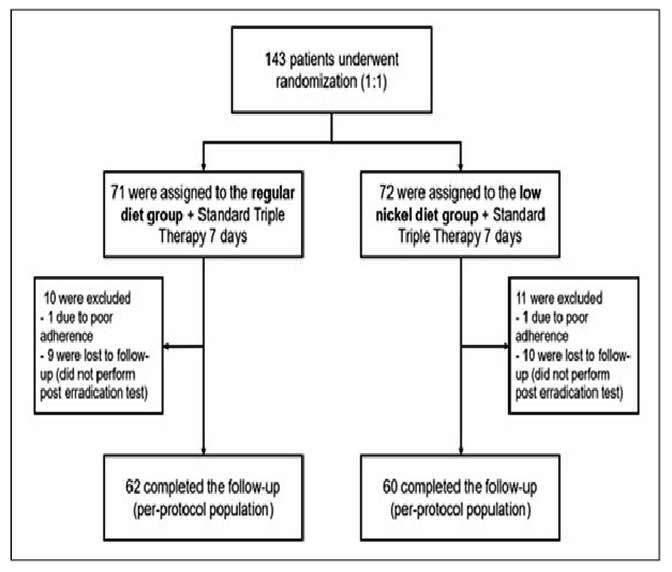
Flowchart of study population.

During the study, the coronavirus disease 2019 (COVID-19) pandemic began, which compromised the follow-up of some patients. Ultimately, 21 patients were excluded; of these, two did not complete the treatment (owing to side effects or poor adherence), and 19 did not return for consultation to evaluate the eradication of the bacteria (Figure 2).

The *H. pylori* eradication rate per protocol (PP) and intention-to-treat (ITT) were analyzed. In the ITT analysis, cases of unknown outcomes were considered to have eradication failure. Chi-square test was used to compare qualitative variables, whereas quantitative variables were analyzed using the Mann-Whitney U test. All tests considered a bidirectional α (*P*-value) of 0.05 and a 95% confidence interval (CI) and were performed using the R software (R Foundation for Statistical Research, Vienna, Austria).

## RESULTS

A total of 143 patients were evaluated for inclusion in the study; of these, 72 were randomized to the intervention group and 71 to the control group. Table 2 shows the epidemiological characteristics of the included population, demonstrating homogeneity between the groups. The choice of PPI was divided into three drugs (lansoprazole, omeprazole, and esomeprazole) according to availability.

In the PP analysis (Table 3), the diet with low-nickel content achieved higher *H. pylori* eradication (91.7%) than the group with a regular diet (75.8%) (*P*=0.026). Patients without obesity (body mass index <30) also showed statistically significant superiority in *H. pylori* eradication (88.9 %) than obese patients (73.2 %) (*P*=0.038). Age, sex, current smoking status, and PPI use did not show statistically significant differences in the rate of *H. pylori* eradication.

**TABLE 2:** Demographic and clinical characteristics of the patients, according to diet group.

	Low-nickel diet (n=72)	Regular diet (n=71)
Age - Years (mean ± SD)	55.9 (±15.6)	53.8 (±13.5)
Women (%)	62.5	66.2
BMI - kg/m^2^ (mean ± SD)	26.3 (±4.9)	28.7 (±6.8)
Obesity - BMI >30 kg/m^2^ (%)	38.9	28.2
Current smoker (%)	8.3	8.5
PPI used in the scheme		
Lansoprazole - n (%)	39 (54.2)	37 (52.1)
Omeprazole - n (%)	21 (29.2)	29 (40.9)
Esomeprazole - n (%)	12 (16.7)	5 (7.0)

BMI: body mass index; PPI: proton pump inhibitor; SD: standard deviation.

**TABLE 3:** *Helicobacter pylori* eradication (per protocol analysis) according to diet intervention, demographic and clinical characteristics.

	Helicobacter pylori eradication		***P*-value**
	Yes	No	
Total - n (%)	102 (83.6)	20 (16.4)	
Diet, % (95% CI)			0.026
Low-nickel diet (n=60)	91.7 (86.7-98.8)	8.3 (3.3-15.4)	
Regular diet (n=62)	75.8 (66.1-86.4)	24.2 (24.5-34.7)	
Age - Years (mean ± SD)	56.9 (±14.7)	51.2 (±12.2)	0.057
Gender, % (95% CI)			0.129
Men (n=40)	90.9 (84.1-98.4)	9.1 (2.3-16.5)	
Women (n=62)	79.5 (71.8-88.7)	20.5 (12.8-29.8)	
BMI - kg/m^2^, % (95%CI)			0.038
<30 kg/m^2^ (n=81)	88.9 (84.0-96.2)	11.1 (6.2-18.4)	
>30 kg/m^2^ (n=41)	73.2 (61.0-86.5)	26.8 (14.6-40.1)	
Current smoker, % (95% CI)			0.211
Yes (n=10)	7 (70.0)	3 (30.0)	
No (n=112)	95 (84.8)	17 (15.2)	
PPI used in the scheme, % (95%CI)			0.453
Lansoprazole (n=61)	83.6 (75.4-92.4)	16.4 (8.2-25.2)	
Omeprazole (n=46)	87.0 (80.4-97.5)	13.0 (6.5-23.6)	
Esomeprazole (n=15)	73.3 (60.0-98.9)	26.7 (13.3-52.2)	

95%CI: 95% confidence interval; BMI: body mass index; PPI: proton pump inhibitor; SD: standard deviation.

In the ITT analysis (Table 4), only age was associated with *H. pylori* eradication, and the mean age of patients with *H. pylori* eradication was 56.9 years and was 50.0 years for those with treatment failure (*P*=0.006). The eradication rate of *H. pylori* in the low-nickel diet group was 76.4%, whereas that in the regular diet group was 66.2%; however, this difference was not statistically significant.

**TABLE 4:** *Helicobacter pylori* eradication (intention-to-treat analysis) according to diet intervention, demographic and clinical characteristics.

	***Helicobacter pylori* eradication**		***P*-value**
	Yes	No	
Total - n (%)	102 (71.3)	41 (28.7)	
Diet, % (95% CI)			0.199
Low-nickel diet (n=72)	76.4 (68.1-86.7)	23.6 (15.3-34.0)	
Regular diet (n=71)	66.2 (56.3-77.9)	33.8 (23.9-45.5)	
Age - Years (mean ± SD)	56.9 (±14.7)	50.0 (±13.2)	0.006
Gender, % (95%CI)			0.181
Men (n=51)	78.4 (68.6-89.7)	21.6 (11.8-32.8)	
Women (n=92)	67.4 (58.7-77.6)	32.6 (23.9-42.8)	
BMI - kg/m^2^, % (95%CI)			0.118
<30 kg/m^2^ (n=83)	75.8 (68.4-85.0)	24.2 (16.8-33.4)	
>30 kg/m^2^ (n=60)	62.5 (50.0-76.8)	37.5 (25.0-51.8)	
Current smoker, % (95%CI)			0.325
Yes (n=12)	58.3 (41.7-91.0)	41.7 (25.0-74.4)	
No (n=131)	72.5 (65.6-80.7)	27.5 (20.6-35.6)	
PPI used in the scheme, % (95%CI)			0.244
Lansoprazole (n=76)	67.1 (57.9-78.6)	32.9 (23.7-44.4)	
Omeprazole (n=50)	80.0 (70.0-90.4)	20.0 (10.0-30.4)	
Esomeprazole (n=17)	64.7 (47.1-89.1)	35.3 (17.6-59.7)	

95%CI: 95% confidence interval; BMI: body mass index; PPI: proton pump inhibitor; SD: standard deviation.

The low-nickel diet group had 3.41 (1.21-11.40) times more chances of *H. pylori* eradication than the regular diet group (PP analysis), as represented in Table 5. In the ITT analysis, the chance of eradication was 1. 61 times higher, but difference was not statistically significant.

**TABLE 5:** Odds ratio for *Helicobacter pylori* eradication (per protocol and intention-to-treat analysis) of low-nickel diet versus regular diet.

	Odds ratio	95%CI
Per protocol	3.41	1.21-11.40
Intention-to-treat	1.61	0.78-3.48

95%CI: 95% confidence interval.

## DISCUSSION

The overall *H. pylori* eradication rate in this study was 83.6%, with only 75.8% eradication in the regular diet group. In a clinical trial by Felga et al.^4^, with a similar population and the same therapeutic drug regimen used in this study (including treatment time), eradication rates of 88.8% were observed even without dietary restrictions. This relevant difference is probably associated with the growing trend of bacterial resistance that occurred over more than 10 years of difference between the studies and justifies the need for strategies to optimize this treatment. When comparing the present results with findings from our 2010 study involving a similar population, we observed that eradication rates were maintained in the group following the nickel-restricted diet, whereas they were significantly lower in the group on a regular diet, suggesting that a lack of nickel intake could overcome bacterial resistance to macrolides^5^.

In the low-nickel diet group, the *H. pylori* eradication rate (per protocol analyses) was 91.7%. In an Italian pilot study published in 2014 that evaluated 52 patients using triple therapy for seven days, the *H. pylori* eradication was 84% in the nickel-free diet group and only 46% in the nonrestricted diet group (*P*<0.01)^15^. It is important to highlight that in this previous study, the restriction of nickel in the diet was performed for 30 days, and triple therapy was only started on day 15^15^. However, in the present study, dietary restriction was recommended only during the use of antibiotics, which may have reduced the efficacy of this strategy.

An important limitation of our study was the high rate of loss to follow-up (14.6%), which considerably affected the ITT analysis. However, this was mainly because of the COVID-19 pandemic, which made it difficult to schedule appointments, endoscopies, and laboratory collections^15^. Therefore, the dropouts were completely random and not a consequence of our intervention, which might have reduced biased results^16^.

Regarding the use of different proton pump inhibitors, equivalent doses were used to assess the efficacy of each medication, specifically lansoprazole 30 mg, esomeprazole 20 mg, and omeprazole 20 mg. In our study, these were administered twice daily for 7 days. A meta-analysis involving 10,339 participants revealed that proton pump inhibitors, at the mentioned equivalent doses, do not show a significant difference in the effectiveness of treatment for peptic disease during a 4-week follow-up^17^.

On analyzing the variables, we noted the impact of obesity as a factor related to the *H. pylori* eradication rate (73.17% vs 88.88%, *P*=0.038). Other studies also showed that patients with obesity treated with PPI-based triple therapy had a lower eradication rate than that of patients with a normal body mass index^7^. Despite the current study’s demographic data showing a predominance of participants with obesity in the low-nickel group (38%) compared to the regular group (28%), better eradication results were observed in the low-nickel group (91.7% vs 75.8%, *P*=0.026). Therefore, we can infer that there might be a possibility of further increase in eradication rate in the low-nickel group if it had been more homogeneous regarding BMI.

An international multicenter study demonstrated that the rate of eradication failure in smokers is almost double that in nonsmokers^18^. However, when analyzing our sample, we observed that although there was a difference in eradication, with greater eradication in nonsmokers (84.82% vs 70%; *P*=0.211), the difference was not statistically significant. This lack of a significant difference is probably associated with the low percentage of smokers in the sample.

In the ITT analysis, the mean age of patients with *H. pylori* eradication was significantly higher than that of patients with therapeutic failure. Previous studies have suggested that older age may lead to greater atrophy of the gastric mucosa and, consequently, hypochlorhydria, which may facilitate *H. pylori* eradication^19^. In a Chinese study, the *H. pylori* eradication rate was 4.58 times higher in those aged 40 years or older (*P*=0.003)^20^.

Despite the limitations of our study, we emphasize the relevance of updating current treatment protocols to extend the regimen to 14 days, as this approach has been associated with higher eradication rates^1^ ^,^ ^3^. Moreover, the study was conducted at a single center and, as previously noted, experienced a considerable dropout rate (14.6%), largely due to the COVID-19 pandemic.

## CONCLUSION

*H. pylori* infection remains highly prevalent and is associated with significant morbidity, including peptic ulcers, gastric cancer, and chronic gastritis. In recent years, increased bacterial resistance to antibiotic regimens has been reported, necessitating the development of new strategies to help eradicate the pathogen. Our study demonstrated that a low-nickel diet, as a low-cost and low-risk intervention, may be a relevant strategy for improving the rate of *H. pylori* eradication. To reinforce these results, further studies should be conducted at other centers with a larger number of participants.
